# The dual developmental origin of spinal cerebrospinal fluid-contacting neurons gives rise to distinct functional subtypes

**DOI:** 10.1038/s41598-017-00350-1

**Published:** 2017-04-07

**Authors:** Lydia Djenoune, Laura Desban, Johanna Gomez, Jenna R. Sternberg, Andrew Prendergast, Dominique Langui, Feng B. Quan, Hugo Marnas, Thomas O. Auer, Jean-Paul Rio, Filippo Del Bene, Pierre-Luc Bardet, Claire Wyart

**Affiliations:** 1 Sorbonne Universités, UPMC Univ Paris 06, Inserm, CNRS, AP-HP, Institut du Cerveau et de la Moelle épinière (ICM) - Hôpital Pitié-Salpêtrière, Boulevard de l’hôpital, F-75013 Paris, France; 2 Muséum National d’Histoire Naturelle, Paris, 75005 France; 3 Institut Curie, Paris, 75005 France; 4 Thomas O Auer, Center for Integrative Genomics, Faculty of Biology and Medicine, University of Lausanne, Lausanne, Switzerland

## Abstract

Chemical and mechanical cues from the cerebrospinal fluid (CSF) can affect the development and function of the central nervous system (CNS). How such cues are detected and relayed to the CNS remains elusive. Cerebrospinal fluid-contacting neurons (CSF-cNs) situated at the interface between the CSF and the CNS are ideally located to convey such information to local networks. In the spinal cord, these GABAergic neurons expressing the PKD2L1 channel extend an apical extension into the CSF and an ascending axon in the spinal cord. In zebrafish and mouse spinal CSF-cNs originate from two distinct progenitor domains characterized by distinct cascades of transcription factors. Here we ask whether these neurons with different developmental origins differentiate into cells types with different functional properties. We show in zebrafish larva that the expression of specific markers, the morphology of the apical extension and axonal projections, as well as the neuronal targets contacted by CSF-cN axons, distinguish the two CSF-cN subtypes. Altogether our study demonstrates that the developmental origins of spinal CSF-cNs give rise to two distinct functional populations of sensory neurons. This work opens novel avenues to understand how these subtypes may carry distinct functions related to development of the spinal cord, locomotion and posture.

## Introduction

The cerebrospinal fluid (CSF) is a complex solution circulating around the central nervous system (CNS). It has classically been assumed that the CSF ensures the homeostasis of the CNS^1^. Multiple studies indicate that the CSF also conveys signals affecting the development and output functions of the CNS, such as feeding, sleep, and locomotion^2–6^. These observations suggest that chemical or mechanical cues from the CSF may act on neurons in the brain and spinal cord. Cerebrospinal fluid-contacting neurons (CSF-cNs) are precisely located at the interface between the CSF and the neuronal circuits^7, 8^. In the vertebrate spinal cord, CSF-cNs reside around the central canal^9–15^ and project an apical extension in contact with the CSF^8, 16–18^ and have a locally-projecting axon^14, 19, 20^ that contacts other spinal neurons^12, 15, 21^.

One essential step to understand spinal CSF-cN functions lies in identifying specific markers for these cells. In this regard, the polycystic kidney disease 2 like 1 (PKD2L1) channel also called TRPP3^22^, which belongs to the Transient Potential Receptor (TRP) family, appears as a robust candidate to label CSF-cNs^11, 13, 23, 24^. PKD2L1, originally identified as the sour taste receptor in the taste buds, has been found in the spinal CSF-cNs of mouse^11, 13, 25, 26^, zebrafish and macaques^13^. The opening of the PKD2L1 channel is modulated by variations in pH^27^ and osmolarity^28^. Although the physiological variations of pH and osmolarity in the CSF are not well known, these observations suggest that CSF-cNs could be interoceptors modulated by chemical and/or mechanical cues from the CSF^23^.

There is evidence that spinal CSF-cNs do not constitute a homogeneous population of neurons. In particular, these cells originate from distinct progenitor domains and are specified differentially by several cascades of transcription factors^26, 29–32^. In zebrafish, CSF-cNs are subdivided into the ventral population, referred to as KA”, originating from the progenitor domain p3 and a dorsal population, referred to as KA’, originating from pMN^29, 30^. In mouse, spinal CSF-cNs were recently shown to originate from the p3 and p2 progenitor domains^26^. In addition, some secreted compounds have been previously reported in a restricted number of spinal CSF-cNs, such as the somatostatin^33, 34^ or serotonin^35, 36^.

The zebrafish has emerged as an ideal model organism to study the development^13, 29, 30^, the morphology and physiological roles of CSF-cNs *in vivo* due to its transparency at early stages^12, 15, 37^. Yet, few functional markers of CSF-cNs related to their sensory or secretory functions have been identified in this species. Here, we investigate whether the two types of spinal CSF-cNs defined by distinct developmental origins can be discriminated by morphology, projection on neuronal targets and expression of secreted compounds. Using a quantitative measure of cell shape, we show that ventral and dorsal CSF-cNs have differently shaped apical extensions as well as different axonal projections and consequently project onto distinct neuronal targets within the spinal cord. By electron microscopy (EM), we found that both populations of CSF-cNs exhibit large granular vesicles in accordance with secretory properties. We demonstrate that these two cell types express distinct modulators and peptides: while ventral CSF-cNs transiently express serotonin, dorsal CSF-cNs express the *sst1*.*1* somatostatin paralog. We observed that the Pkd2l1 channel is not required for the differentiation of CSF-cN axonal projections, for the expression of serotonin or somatostatin. Altogether, our results show that spinal CSF-cNs constitute two distinct functional cell types that differ in apical and axonal morphology, neuronal targets within the spinal cord as well as in the transient expression of secreted compounds.

## Results

### CSF-cNs are neurosecretory cells with an apical extension into the central canal

**Figure 1:**
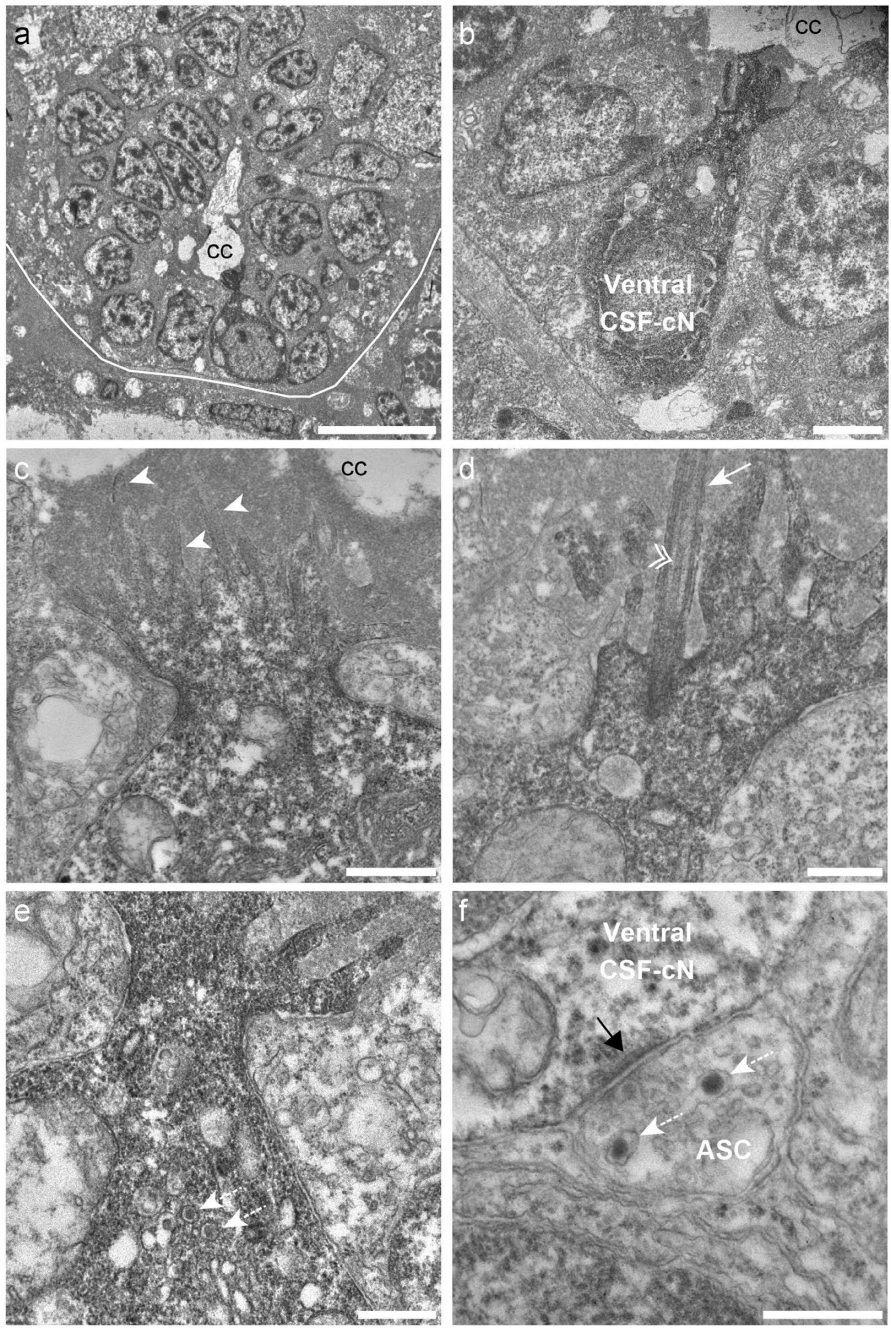
Ventral CSF-cNs exhibit an apical extension composed of microvilli and a kinocilium in the spinal cord. (**a**) Transverse section of the spinal cord showing restricted deposition of DAB in a ventral CSF-cN. (**b**) Overall view of a DAB^+^ ventral CSF-cN contacting the central canal (cc) and surrounded by ependymal cells. (**c**) Ventral CSF-cNs project at the apical pole an extension toward the central canal bearing several microvilli (arrowheads). (**d**) Within this extension is located a cilium (arrows) with two central microtubule along the axoneme (double arrowhead), suggesting a motile cilium. Large granular vesicles (LGV) are observed in the cytoplasm (**e**, dotted arrows) and axo-somatic synaptic contacts in the basal pole (**f**, ASC). Note the symmetry of the synaptic contact (black arrow) is reminiscent of an inhibitory synapse. Note the presence of LGV in the axon (**f**, dotted arrows). Scale bar = 10 μm (**a**), 2 µm (**b**), 1 μm (**c**), 500 nm (**d**,**e**) and 400 nm (**f**).

**Figure 2:**
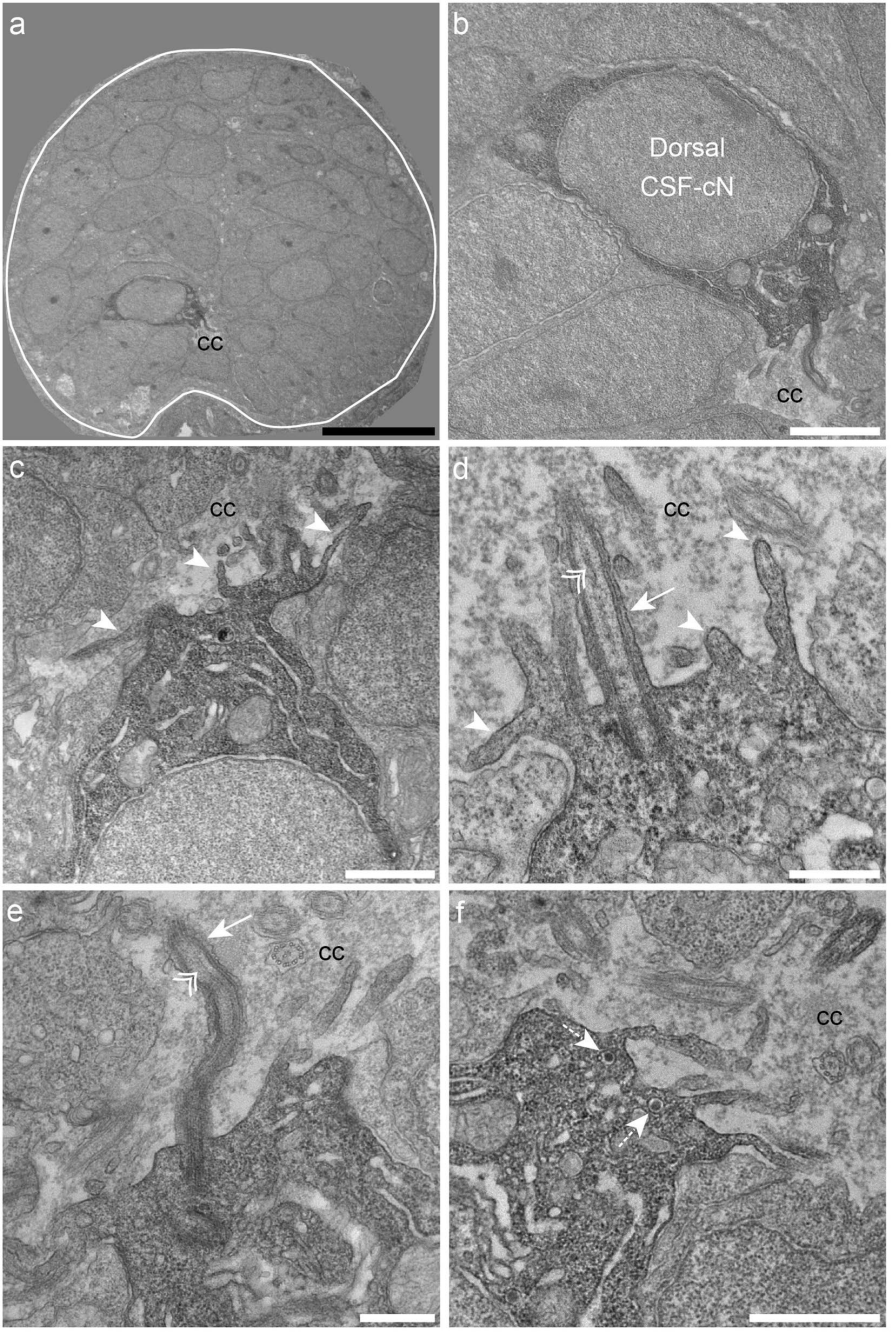
Dorsal spinal CSF-cNs also exhibit ultrastructural properties of sensory neurons. (**a**) Transverse section of the spinal cord showing restricted deposition of DAB in a dorsal CSF-cN. (**b**) Overall view of a DAB^+^ dorsal CSF-cN contacting the central canal (cc). (**c**,**d**) Dorsal CSF-cNs bear at the apical pole multiple microvilli (arrowheads). (**d**,**e**) In the apical pole is located a cilium (arrow) with two central microtubule singlets along the axoneme (double arrowhead), reminiscent of a motile cilium. (**f**) Dorsal CSF-cN also exhibit LGV distributed in the cytoplasm (dotted arrows). Scale bar = 10 μm (**a**), 2 μm (**b**), 1 µm (**c**,**f**) and 500 nm (**d**,**e**).

We characterized CSF-cN ultrastructure in transverse sections of the 2.5 days post fertilization (dpf) zebrafish spinal cord by transmission electron microscopy (TEM). Without specific markers, we observed cells around the central canal that bore an apical extension with microvilli (Supplemental Fig. 1). To demonstrate these cells were CSF-cNs, we transiently expressed the engineered genetically-encoded peroxidase APEX2 under the *pkd2l1* promoter (see Materials and Methods, Supplemental Fig. 2). In APEX2-TagRFP^+^ larvae, we performed diaminobenzidine (DAB) staining (see Material and Methods, and Supplemental Fig. 3). DAB was detected only within CSF-cNs in the spinal cord, making the cells appear darker (Supplemental Fig. 3b). The specific DAB staining (Figs 1 and 2) revealed that CSF-cNs were either round (Figs 1a and 2a-b) or pear-shaped (Fig. 1b). Ventral and dorsal CSF-cNs bear an apical extension with several microvilli directed toward the lumen of the central canal (Figs 1c and 2c). Among actin-based microvilli, we observed, as previously described in one cell^37^, a single cilium directed toward the CSF (Fig. 1d, n = 12 ventral CSF-cNs, Fig. 2d, n = 3 dorsal CSF-cNs). This cilium bears two central microtubule singlets along the axoneme (Figs 1d and 2d-e), often seen in a kinocilium^38, 39^. In APEX2^+^ CSF-cNs, we observed large granular vesicles (Figs 1e and 2f), suggesting that these cells either release or uptake peptides or neuromodulators into or from the CSF. Interestingly, we observed one axo-somatic symmetric synaptic contact at the basal pole of a CSF-cN (Fig. 1f), suggesting that CSF-cNs receive inhibitory synaptic inputs. Indeed, asymmetric synapses usually contain glutamate, are non-GABA immunoreactive and are therefore considered excitatory, while symmetric synapses contain GABA and are considered inhibitory^40–42^. Altogether, our TEM data show that both populations of CSF-cNs exhibit properties of sensory and secretory cells.

### CSF-cNs display different shapes of apical extensions and axonal projections

**Figure 3:**
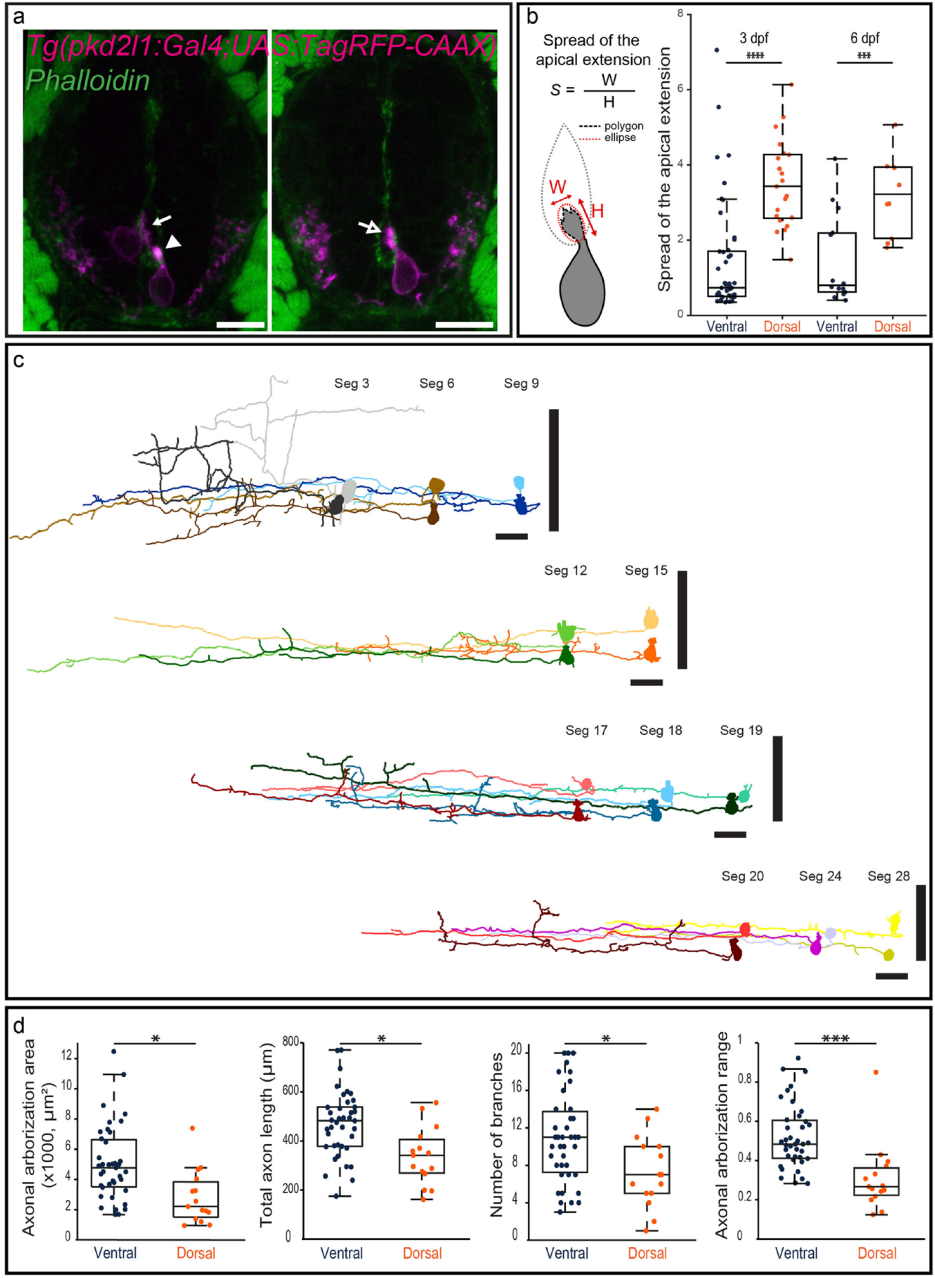
Morphological analysis of CSF-cNs reveals heterogeneous shapes of apical extension and axonal projections. (**a**) Transverse sections showing ventral and dorsal TagRFP-CAAX^+^ CSF-cNs (magenta) at 3 dpf reflecting the diversity of morphologies of the apical extension. The apical extension of all dorsal CSF-cNs spreads along the central canal border (arrow) while most ventral CSF-cNs (86.7%) form compact extensions (arrowhead). The small remaining subpopulation of ventral CSF-cNs exhibits the typical spread of dorsal apical extensions (arrow with empty head; Phalloidin staining, green). (**b**) Schematics of the analysis of the apical extension performed on each cell and statistical analysis comparing the size of the apical extension between ventral and dorsal CSF-cNs at 3 dpf (n = 45 versus 21) and 6 dpf (n = 14 versus 10). The apical extension of dorsal CSF-cNs extends more than for ventral CSF-cNs (two-sample t-tests, p < 5 · 10^−7^) and this difference persists at later stages (6 dpf, p < 0.002). (**c**) The reconstruction from dorsal (light shade) and ventral (dark shade) CSF-cNs from different segments (Seg) illustrates the diversity of axonal morphologies CSF-cNs between the two types along the spinal cord (n = 11 for each type). Vertical black bars represent the dorso-ventral limits of the spinal cord. Cells are positioned according to their dorso-ventral (D-V) position with dorsal edge set to 1 and ventral to 0. (**d**) Comparison of ventral and dorsal CSF-cNs for axonal arborization area, total axon length, number of branches and axonal arborization dorso-ventral range (n = 39 and 15 cells respectively). Ventral CSF-cNs have a wider axonal arborization (p < 0.003), a longer axon (p = 0.0014), reach more ventral domains of the spinal cord (p < 9 · 10^−4^), and cover a larger dorso-ventral (D-V) range (p < 2 · 10^−4^) with more axonal branches (p < 0.02). Two-sample t-tests were performed to compare the two populations. Scale bar = 10 μm (**a**) and 20 µm (**c**).

As a quantitative analysis of CSF-cN morphology is difficult using serial EM, we turned to fluorescence for a quantitative comparison of the apical extension and the axonal projections of dorsal and ventral CSF-cNs. First, we tested whether these cells differ in the morphology of their apical extension. We investigated the shape of the CSF-cN apical extension by labeling the membrane of CSF-cNs in *Tg*(*pkd2l1*:*Gal4*;*UAS*:*TagRFPCAAX*;*cmlc2*:*eGFP*)*icm22* larvae or F-actin itself in *Tg*(*pkd2l1*:*Gal4*;*UAS*:*LifeAct-GFP*;*cryaa*:*V*)*icm28* larvae or larvae transiently expressing (*pkd2l1*:*Gal4*) and (*UAS*:*LifeAct-TagRFP*;*cryaa*:*C*). Using these different approaches, we found that the shape of the apical extension differs between ventral and dorsal CSF-cNs at 3 and 6 dpf (Fig. 3a,b and Material and Methods). The apical extension of dorsal CSF-cNs (Fig. 3a,b) was more extended along the border of the central canal than for ventral cells (Fig. 3a,b). The apical extension in ventral CSF-cNs was overall more compact with a small proportion displaying a more extended apical extension similar to dorsal CSF-cNs (13.3%, n = 6 out of 45 ventral CSF-cNs, Fig. 3a, empty arrow). Next, to assess whether ventral and dorsal CSF-cNs differ in their axonal projections, we injected the (*pkd2l1-TagRFP*) DNA construct to label single cells (Supplemental Fig. 4a) and reconstructed their axon (Fig. 3c, Supplemental Fig. 4b,c). All CSF-cN axonal projections were ventral, ipsilateral and ascending, yet they were heterogeneous along the rostrocaudal axis of the spinal cord (n = 54, Fig. 3c). We measured significant differences between the axonal projections of the two populations of CSF-cNs in terms of the area of the axonal arborization, the length of the axon, the dorso-ventral coverage within the spinal cord and the number of axonal branches (Fig. 3d). The larger axonal length, arborization and number of branches in ventral CSF-cNs compared to dorsal cells suggest these ventral cells possibly differentiate before dorsal ones. Nonetheless, the fact that the dorsal and ventral populations of CSF-cNs project on different domains along the dorso-ventral axis of the spinal cord suggests that these two populations may target different neuronal types within the ventral spinal cord.

### Distinct neuronal targets for ventral and dorsal CSF-cNs within the spinal cord

**Figure 4:**
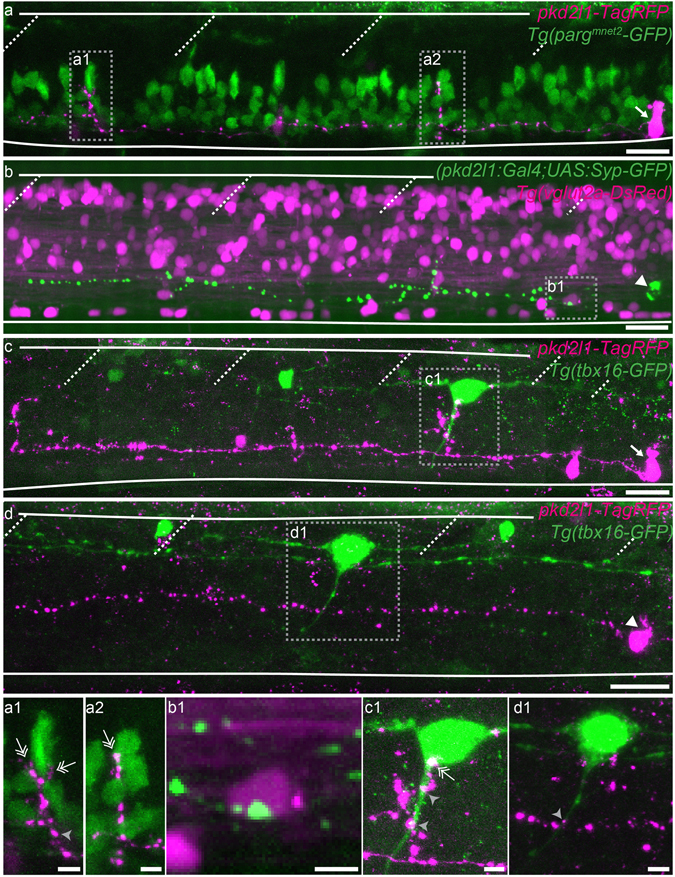
Ventral and dorsal CSF-cNs project onto distinct neuronal populations. (**a**–**d**) Z projection stacks showing contact from ventral and dorsal CSF-cNs onto different spinal targets. (**a**) Lateral view of a ventral CSF-cN (magenta, arrow) contacting 2 CaP motor neurons (identified based on their location within the segment) labelled in green in the *Tg*(*parg* ^*mnet2*^ *-GFP*) transgenic line (**a1**,**a2**, double headed arrows). (**b**) Dorsal CSF-cN (green, arrowhead) contacting a putative V0-v interneuron (magenta, based on its dorso-ventral and lateral location) in the *Tg*(*vglut2a*:*DsRed*) transgenic line. (**c**,**d**) Ventral (**c**, arrow) and dorsal (**d**, arrowhead) CSF-cNs (magenta) contact CoPA sensory interneurons (green) labelled in the *Tg*(*tbx16-GFP*) transgenic line. Boxes with close-ups highlight contacts between the CSF-cN and its target. Scale bar = 20 µm (**a**–**d**) and 5 µm (**a1**–**d1**).

Differences in the axonal projection of ventral and dorsal CSF-cNs suggest these cells may project onto distinct targets within the spinal cord. Our lab recently demonstrated that CSF-cNs project onto caudal primary (CaP) motor neurons and commissural primary ascending (CoPA) sensory interneurons within the escape circuit^43^ as well as onto V0-v interneurons within the slow swimming circuit^15^. We investigated which CSF-cN type projects onto these targets using mosaic labeling in transgenic lines labeling CaP (*Tg*(*parg* ^*mnet2*^ *-GFP*)^43, 44^), V0-v (*Tg*(*vglut2a*:*lox*:*DsRed*:*lox*:*GFP*)^15, 45^) or CoPA (*Tg*(*tbx16-GFP*)^43, 46^). We observed that only ventral CSF-cNs form the stereotypical basket structure around the soma of CaP motor neurons (Fig. 4a, n = 7 out of 8 ventral CSF-cNs found projecting onto CaP and 0 out of 5 dorsal CSF-cNs). In contrast, we found only dorsal CSF-cNs contacting ventrolateral glutamatergic cells, putatively V0-v based on their location and our previous findings^15, 47–49^ (Fig. 4b, n = 3 out of 3 dorsal CSF-cNs and 0 out of 5 ventral CSF-cNs). Interestingly, we observed that both ventral and dorsal CSF-cNs project onto CoPA sensory interneurons (Fig. 4c,d, n = 4 dorsal and 5 ventral CSF-cNs). Altogether, our observations demonstrate a complex connectivity pattern from CSF-cNs onto their neuronal targets in the spinal cord. While some targets receive projections from both CSF-cN types (such as CoPA sensory interneurons), others seem to only receive inputs from either ventral (CaP motor neurons) or dorsal (ventrolateral glutamatergic neurons, most likely V0-v interneurons) CSF-cNs.

### Investigation of secreted compounds expressed in dorsal and ventral CSF-cNs

We tested whether ventral and dorsal CSF-cNs differentially express secreted compounds previously reported in a restricted number of CSF-cNs, namely somatostatin^14, 21, 33, 34^ and serotonin^35, 36^. By taking advantage of *pkd2l1* expression in CSF-cNs^13^, we used the *Tg*(*pkd2l1*:*GCaMP5G*)*icm07* transgenic line to selectively target CSF-cNs^37^. We demonstrated the specificity of this line using fluorescent *in situ* hybridization (FISH) for *pkd2l1* mRNA coupled to GFP immunohistochemistry (IHC) to amplify the endogenous GCaMP5G signal from 24 hours post fertilization (hpf) to 5 dpf (Supplemental Fig. 5).

**Figure 5:**
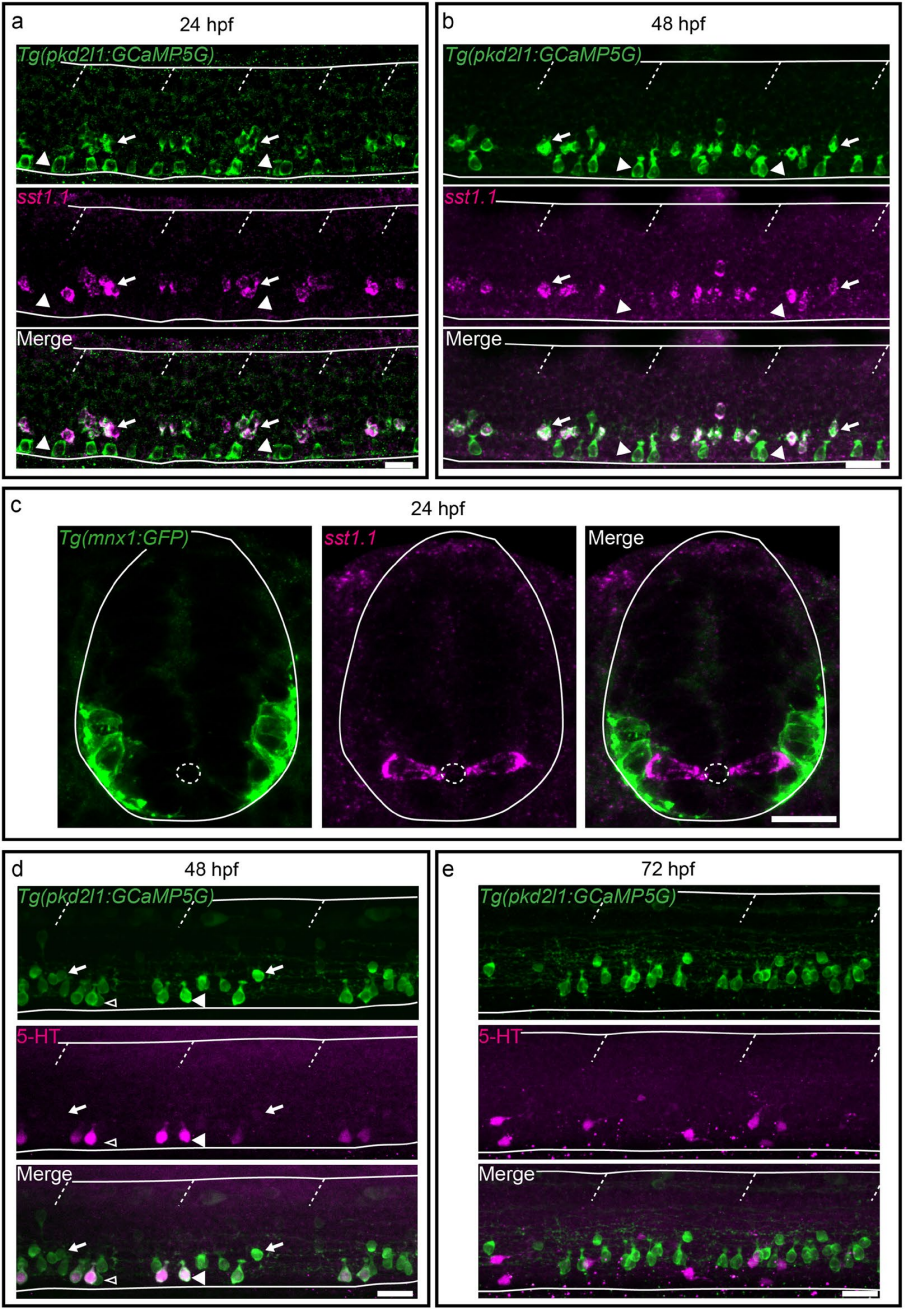
Secreted factors distinguish dorsal and ventral CSF-cNs: while dorsal express the somatostatin paralog *sst1*.*1*, ventral CSF-cNs express 5-HT. (**a**–**c**) Lateral views of the spinal cord show that *sst1*.*1* expression is restricted to dorsal CSF-cNs (arrows, FISH for *sst1*.*1* (magenta) coupled to GFP IHC (green) on *Tg*(*pkd2l1*:*GCaMP5G*)) (**a**,**b**) and *Tg*(*mnx1*:*GFP*) (**c**) embryos and larvae at 24 hpf (**a**,**c**) and 48 hpf (**b**). (**c**) Transverse sections show that *sst1*.*1* (magenta) is not expressed in motor neurons (green) as previously suggested (Devos *et al*.^52^). (**d**,**e**) IHC for 5-HT (magenta) and GFP (green) on *Tg*(*pkd2l1*:*GCaMP5G*) transgenic larvae at 48 hpf (**d**) and 72 hpf (**e**). (**d**) At 48 hpf, most ventral CSF-cNs express 5-HT (arrowhead, compared to negative cells shown with empty arrowhead). Note that dorsal CSF-cNs (arrows) are not labelled by 5-HT. (**e**) At 72 hpf, ventral CSF-cNs are not serotoninergic anymore in the rostral part of the spinal cord. Horizontal lines represent the limits of the spinal cord and slash dashed lines represent somite boundaries. Small dotted ellipses represent the limit of the central canal. Scale bars = 20 μm.

In zebrafish, somatostatin immunoreactivity has previously been reported^12^, but 6 different paralogs exist (*sst1*.*1*, *sst1*.*2*, *sst2*, *sst3*, *sst5* and *sst6*)^50, 51^. Testing the expression of all of them, we only detected expression of *sst1*.*1* in CSF-cNs. Expression of *sst1*.*1* was observed only in dorsal CSF-cNs (Fig. 5a,b, arrows) in the rostral part of the spinal cord, from segment 1 to 13 at 24, 48, 72 hpf and in the adult spinal cord (data *not shown*). Since *sst1*.*1* had been reported as expressed transiently in motor neurons from 19 hpf until 55 hpf^52^, we showed using FISH for *sst1*.*1* with GFP IHC in 24 hpf *Tg*(*mnx1*:*GFP*) embryos^53^ that *sst1*.*1* expression was excluded from motor neurons (Fig. 5c). Altogether, our results show that *sst1*.*1* is expressed in dorsal CSF-cNs and absent in ventral ones at early stages and restricted as well to a subpopulation of CSF-cNs in the adult. Next, we tested whether zebrafish CSF-cNs were serotoninergic by performing double IHC for 5-HT and GFP on *Tg*(*pkd2l1*:*GCaMP5G*)*icm07* embryos and larvae from 24 hpf to 72 hpf. At 24 hpf, no 5-HT immunostaining was detected in the zebrafish spinal cord (*data not shown*). At 48 hpf, we detected 5-HT in ventral CSF-cNs (Fig. 5d, arrowheads) in the rostral part of the spinal cord (from segment 1 to 24). This expression was restricted to a subset of ventral CSF-cNs (Fig. 5d). The proportion of 5-HT^+^ CSF-cNs at 48 hpf decreased along the rostrocaudal axis of the spinal cord (segments 3 to 6: 88.6 ± 16.0%, 96 cells; segments 10 to 13: 73.2 ± 27.3%, 100 cells; segments 23 to 26: 31.2 ± 33.3%, 94 cells). At 3 dpf, although 5-HT was still expressed along the entire spinal cord in other cells, the staining was absent in CSF-cNs in the rostral spinal cord (Fig. 5e). In conclusion, dorsal CSF-cNs express *sst1*.*1* while a subpopulation of ventral CSF-cNs population is transiently serotoninergic. Hence, these two populations originating from different progenitor pools differentiate into distinct cell types expressing specific secreted compounds.

### *pkd2l1* null mutation does not impact on the differentiation of spinal CSF-cNs

We previously showed that the PKD2L1 channel is expressed in spinal CSF-cNs across multiple vertebrate species^13^ and that the channel appears necessary to mediate detection of spinal bending by CSF-cNs in the larva^37^. In *pkd2l1* mutants, we observed defects in locomotion that we interpreted as being caused by the lack of sensory responses in CSF-cNs^37^. To strengthen the conclusions of this previous study, we sought to precisely assess whether CSF-cNs develop properly in the *pkd2l1* mutants. We found that in mutants, CSF-cNs were still GABAergic, ventral ones were still serotoninergic, that the number of CSF-cNs was not impacted, and that the morphology of their axonal projections was not different from WT (Supplemental Fig. 6). We also wanted to determine whether the functional connection between ventral CSF-cNs and CaPs^43, 54^ was impacted and found that light-mediated activation of mutants CSF-cNs was still able to induce monosynaptic inhibitory postsynaptic currents in CaPs (Supplemental Fig. 7). Altogether, our results show that *pkd2l1* is not required for CSF-cN differentiation.

## Discussion

Spinal CSF-cNs were previously shown to originate from distinct progenitor domains characterized by distinct pools of transcription factors in the embryo^26, 29, 30^. Here we demonstrate that these two domains give rise to two cell types of CSF-cNs with probably distinct functional properties at the larval stages (Supplemental Fig. 8). First, the morphology of these two cell types differs both at the apical extension level as well as at the axonal level. Furthermore, we show that these differences in axonal projections lead to differences in neuronal targets reached in the spinal cord. Second, our ultrastructure data indicate that both ventral and dorsal CSF-cNs bear dense granules under the apical extension, suggesting possible roles in release or uptake of compounds from the CSF. We show that ventral and dorsal populations express distinct secreted factors, in particular 5-HT and *sst1*.*1*, during development. Finally we show that Pkd2l1 is not necessary for the differentiation of CSF-cNs. Altogether, our results demonstrate that the two cell types of CSF-cNs previously identified with different developmental origins segregate into distinct populations bearing specific functional properties.

Due to their peculiar apical extension contacting the CSF, it had been hypothesized that CSF-cNs may release compounds into the CSF^7, 55^. The presence of large granular vesicles (LGV) within CSF-cNs in our EM data, combined with expression of *sst1*.*1* and 5-HT for dorsal and ventral CSF-cNs respectively, reinforces this hypothesis.

Previous works have shown that CSF-cNs are chemosensory^11, 24, 56, 57^ as well as mechanosensory cells^37, 43, 58^. In zebrafish larvae, we previously showed that dorsal CSF-cNs respond to lateral bending of the spinal cord^37^ while ventral CSF-cNs respond to longitudinal contractions^43^. The difference in shape of the apical extension between dorsal and ventral CSF-cNs may reflect a structural difference relevant for either their mechanosensory functions (direction of maximum sensitivity to bending of the tissue or CSF flow) or their secretory functions (with possibly larger surface area for dorsal CSF-cNs).

There are indications that spinal CSF-cNs harbor different morphologies in other species as well. In rat, spinal GABAergic CSF-cNs were first classified into three subtypes according to the shape of their soma^59^. Four morphological types of CSF-cNs were then described based on the shape of their soma, their axonal projection, and on the expression of the peptide Met-Enk-Arg-Gly-Leu^60^. These indications together with our data reveal a high level of heterogeneity among spinal CSF-cNs. Future studies will investigate the role of CSF-cN structural differences in sensory and secretory functions.

Using a single cell labelling approach, we show that ventral CSF-cNs have on average a longer and broader axonal arborization and cover a higher dorso-ventral spinal cord range with more axonal branches than dorsal CSF-cNs. These differences in axonal projections suggest the two CSF-cN populations might have distinct targets within the spinal cord. We had shown that CSF-cNs form active GABAergic synapses onto glutamatergic descending V0-v interneurons^15^ as well as CaP primary motor neurons and CoPA sensory interneurons^43^. Here we show that only ventral CSF-cNs form the characteristic basket-like contact onto CaP soma while we only found dorsal CSF-cNs contacting ventrolateral glutamatergic neurons, most likely V0-v interneurons^47–49^ previously shown to receive inputs from CSF-cNs^15^. Interestingly CoPA sensory interneurons received innervation from both dorsal and ventral CSF-cNs. Our results suggest that targets of the slow swimming circuits receive innervation from dorsal CSF-cNs while targets of the fast swimming circuit receive innervation from ventral CSF-cNs, and sensory interneurons involved in processing feedback receive innervation from both ventral and dorsal CSF-sNs. Further investigations will be necessary to establish whether other targets of the slow and fast swimming circuits follow the same dichotomy, receiving inputs from dorsal and ventral CSF-cNs respectively.

Multiple markers have been found in subsets of CSF-cNs across vertebrate species: somatostatin^12, 14, 21, 33, 34^, dopamine^61–64^ and serotonin^35, 36, 65^. The expression of most markers was reported in a restricted fraction of CSF-cNs. Here we tested whether some of these markers showed restricted expression patterns among CSF-cNs. We found that ventral CSF-cNs were at 48 hpf transiently serotoninergic as previously described in other species^35, 36, 65^ in accordance with findings from Montgomery *et al*.^66^, in zebrafish. In addition, we demonstrated that, even at 48 hpf, not all ventral CSF-cNs expressed 5-HT. This suggests that there may be different subtypes of ventral CSF-cNs based on 5-HT expression. Moreover, Montgomery *et al*.^66^, also showed that the rate-limiting enzyme involved in 5-HT synthesis *tryptophan hydroxylase 1* a (*tph1a*)^67, 68^ is expressed in the ventral spinal cord at 24 and 48 hpf suggesting that ventral CSF-cNs could constitute a transient source of serotonin at early stages. One possible explanation for the disappearance of 5-HT in ventral CSF-cNs at later stages could be the emergence of descending fibers from 5-HT^+^ neurons originating from the brainstem raphe nuclei from 48 hpf onwards^67^. 5-HT from descending fibers has been previously shown in mammals to suppress the monoaminergic expression of CSF-cNs^69–71^. The physiological relevance of the transient 5-HT expression among this ventral population of CSF-cNs remains to be elucidated.

Regarding the origin of somatostatin in zebrafish CSF-cNs, we found that among the six SST paralogs, *sst1*.*1* was the only one expressed. We demonstrated that the expression of this gene is specific and restricted to the dorsal population of CSF-cNs, identifying for the first time a specific marker of dorsal CSF-cNs. We also demonstrated that this peptide is excluded from motor neurons contrary to what previous studies suggested^52^. The physiological relevance of *sst1*.*1* expression by dorsal CSF-cNs is not known. In mammals and lamprey, it has been shown that somatostatin can reduce locomotor frequency^58, 72, 73^. The release of SST by dorsal CSF-cNs could impact the frequency of locomotor events along with the release of neuropeptides of the UII family, *urp1* and *urp2* by ventral CSF-cNs only^74^ as previously suggested by the presence in coho salmon of a UII-like immunoreactive ventral population of CSF-cNs and a distinct somatostatinergic one^75^. Taken together, our results confirm that ventral and dorsal CSF-cNs express distinct peptides and neuromodulators. The transient nature of the expression of 5-HT suggest that it could have developmental roles to be further investigated.

## Material and Methods

### Animal care

Zebrafish (*Danio rerio*) adults and larvae were maintained and raised on a 14/10 hour light cycle. Fish lines used in this study are referenced in Table 1. Water was regulated at 28.5 °C, conductivity at 500 μS and pH at 7.4. All embryos and larvae under 6 dpf were anesthetized in 0.02% tricaine methane sulfonate (MS 222) (Sandoz, Levallois-Perret, France) and euthanized in 0.2% MS 222 prior to fixation. Experimental and animal protocols were approved by the Institut du Cerveau et de la Moelle Épinière in agreement with the French National Ethics Committee (Comité National de Réflexion Éthique sur l’Éxpérimentation Animale; Ce5/2011/056) and European regulations.Genotype protocol for the *pkd2l1* ^*icm02*^ mutants was described in ref. 37 (Böhm *et al*. 2016).

**Table 1:** Transgenic lines used in our study.

Transgenic/mutant	Labelling in the spinal cord	Original publication
*Tg(pkd2l1:GCaMP5G)icm07*	CSF-cNs	37
*Tg(pkd2l1:GCaMP5G)icm07;pkd2l1* ^*icm02*^	CSF-cNs	37
*Tg(mnx1:GFP)*	Motor neurons	53
*Tg(pkd2l1:Gal4)icm10*	CSF-cNs	15
*Tg(UAS:TagRFP-CAAX;cmlc2:eGFP)icm22*	Non applicable	This study
*Tg(UAS:LifeAct-GFP;cryaa:V)icm28*	Non applicable	This study
*Tg(parg* ^*mnet2*^ *-GFP)*	Motor neurons	44
*Tg(tbx16-GFP)*	CoPAs	46
*Tg(vglut2a:lox:DsRed:lox:GFP)*	Glutamatergic interneurons	45

### Generation of transgenic lines

In order to generate the *Tg*(*UAS*:*TagRFP-CAAX*;*cmlc2*:*eGFP*)*icm22* line, the TagRFP-CAAX sequence was amplified using a TagRFP-forward (5′-CCCGGGATCCACATGGTGTCTAAGGGCGAAG-3′) and reverse primer (5′-GATCGCGGCCGCTCAGGAGAGCACACACTTGCAGCTCATGCAGCCGGGGCCACTCTCATCAGGAGGGTTCAGCTTATTAAGTTTGTGCCC-3′) and inserted into the *pME-MCS* vector^76^ via BamHI/NotI restriction digestion. The resulting *pME-TagRFP-CAAX* vector was recombined via a Gateway reaction (MultiSite Gateway Three-Fragment Vector Construction Kit) with *p5E-4nrUAS* ^77^, *p3E-pA* and *pDest-Tol2*; *cmlc2*:*eGFP* ^76^ resulting in (*p4nrUAS*:*TagRFP-CAAX-pA-Tol2*;*cmcl2*:*eGFP*). Using a similar approach, the *pME_LifeAct-GFP* plasmid was generated from the plasmid *pCS2_LifeAct-GFP* (kind gift from Nicolas David) using the SP6 promoter sequencing primer (5′-ATTTAGGTGACACTATAG-3′) and inserted into the pME_MCS vector via a BamHI/NotI restriction digestion. The final three-way gateway reaction used the *pME_LifeAct-GFP*, *pDest_cryaa*:*V* and *p5*′*E_polyA* plasmids to generate the (*UAS*:*LifeAct-GFP*;*cryaa*:*V*) construct. Microinjection of these plasmids was performed with Tol2 mRNA (25 ng/µl) following standard protocols. Transgenic founder fish *Tg*(*UAS*:*TagRFP-CAAX*;*cmlc2*:*eGFP*)*icm22* where screened based on GFP expression in the heart. These two lines were also screened based on transactivation of their respective transgene when crossed with various Gal4 lines.

### Plasmid design

In order to investigate the CSF-cNs ultrastructure, we took advantage of the engineered peroxidase APEX2^78^. We generated a three-fragment Gateway recombining reaction (Invitrogen, Carlsbad, CA, USA) to design the (*UAS*:*APEX2-TagRFP*) construct. This plasmid was injected into single-cell stage *Tg*(*pkd2l1*:*Gal4*)*icm10* embryos at 60 ng/µl or co-injected with the (*pkd2l1*:*Gal4*)*icm10* construct into *Tg*(*pkd2l1*:*GaMP5G*)*icm07* singe-cell stage embryo. To generate the (*UAS*:*LifeAct-TagRFP*;*cryaa*:*C*) DNA construct, we extracted the coding sequence of the tagged protein LifeAct-TagRFP from the plasmid (*mTagRFP-T- LifeAct-7*) (Addgene #54586, kind gift from Michael Davidson) by PCR using a forward (5'-GGGGACAAGTTTGTACAAAAAAGCAGGCTAGATCTCTGCCACCATGGGCGTGGCCGACTTGATC-3') and a reverse (5'-GGGGACCACTTTGTACAAGAAAGCTGGGTACTAGTTTACTTGTACAGCTCGTCCATGCC-3') primer. LifeAct-TagRFP was then inserted into the plasmid *pDONR221* by a BP reaction to produce the *pME_LifeAct-TagRFP* plasmid and a three-way gateway reaction was performed using *pME_LifeAct-TagRFP*, *pDest_cryaa*:*C*, *p5*′*E_10XUAS* and *p3*′*E_polyA* plasmids to produce the (*UAS*:*LifeAct-TagRFP*;*cryaa*:*C*) construct.

### Electron microscopy

To label CSF-cNs for electron microscopy, we followed the procedure described in refs 37, 78 and Supplemental Figs 2 and 3. 2.5 dpf larvae selected for TagRFP expression in CSF-cNs were primarily anaesthetized in 0.02% tricaine methane sulfonate (MS 222) then euthanized in 0.2% tricaine prior to fixation in 2% glutaraldehyde in 100 mM sodium cacodylate buffer pH 7.4 to which 2 mM of CaCl_2_ was added for 45 min. Whole embryos were then rinsed in the same buffer 5 × 2 min each. Functional aldehyde excess was blocked by treating embryos for 5 min with 20 mM glycine in the sodium cacodylate buffer. Other rinses in buffer (5 × 2 min) were then carried out. The APEX2 peroxidase was further revealed in diaminobenzidine (DAB, 0.5 mg/ml) to which 10 mM hydrogen peroxide (30%) was added (see Lam *et al*.), in 100 mM cacodylate buffer. After 5 min, the reaction was stopped by rinsing embryos twice in cold buffer before post fixation in 2% osmium tetroxide (OsO_4_) in the same buffer for 45 min. Embryos were then rinsed in cold distilled water, stained in 2% uranyl acetate in distilled water overnight in a cold chamber. Animals were returned to room temperature, rinsed in distilled water, then dehydrated in a graded series of ethanol, cleared in acetone, and embedded in epoxy resin (EMBed812, Electron Microscopy Science, France). Embryos were oriented coronally in resin molds cured at 60 °C for 48 hr. Whole embryos were imaged using a Leica DMRB microscope to assess the presence of DAB positive CSF-cNs and determine their location within the animals. Embryos were first cut in 1 µm semi-thin sections with an ultramicrotome (Ultracut E, Leica). Sections were picked up every µm, stained with toluidine blue. Semi-thin sectioning was performed until the first level of DAB^+^ CSF-cN cell appears. Then, serial ultra-thin sections (~70 nm thick) were collected onto copper grids (about 8 sections per grid). 12 ventral CSF-cNs, and 3 dorsal CSF-cNs were analyzed. They were contrasted in urany-less solution (Delta Microscopies, France) for 1–2 min, rinsed in distilled water, dried for at least 1 hr. Observations were made with an Hitachi HT 7700 electron microscope operating at 70 kV. Electron micrographs from DAB^+^ CSF-cNs were taken at low (×6200), medium (×22,000) and high (×53,000) magnifications, using an integrated AMT XR41-B camera (2048 × 2048 pixels). In all the images displayed, dorsal is up and rostral is left.

### Analysis of the apical dendritic extension of CSF-cNs

To image the apical dendritic extension at a high resolution, we used different labeling strategies relying on transverse sections of the spinal cord. At 3 dpf, we used the stable *Tg*(*pkd2l1*:*Gal4*;*UAS*:*TagRFP-CAAX*;*cmlc2*:*eGFP*) transgenic line with membrane TagRFP and the transient expression of (*pkd2l1*:*Gal4*)^15^ and (*UAS*:*LifeAct-TagRFP*;*cryaa*:*C*) DNA constructs injected into *Tg*(*pkd2l1*:*GCaMP5*)*icm07* eggs at the one cell stage to label F-actin with LifeAct^79^. At 6 dpf, we took advantage of the stable *Tg*(*pkd2l1*:*Gal4*;*UAS*:*LifeAct-GFP*;*cryaa*:*V*)*icm28* line. 3 and 6 dpf larvae were screened for expression in CSF-cNs and fixed using 4% paraformaldehyde (PFA) during 4 hours at 4 °C and immunostained as described below. We sliced 50 µm-thick transverse sections on larvae mounted in 3% low melting point agarose using a vibratome (HM 650 V Microtome, Thermo Scientific). For each cell, Z stacks (step size 0.25 µm) were acquired to image the entire apical extension and maximum projection was performed in Fiji^80^. The shape of the apical extension (S) was calculated as the ratio of the width (W, basal extension along the central canal) over the height (H, vertical extension within the central canal). W and H were extracted by drawing a polygon around the apical extension using the polygon tool in Fiji and estimated by the parallel and perpendicular axis, respectively, of the best fitting ellipse (Fig. 4b).

### Single cell labeling

To assess whether *pkd2l1* mutation led to a disruption of CSF-cN axonal refinement, we injected at 25 ng/μl the (*pkd2l1-TagRFP*) construct generated with a three-fragment Gateway recombineering reaction into single-cell stage embryos from *pkd2l1* ^*icm02*/+^ incrosses (see ref. 15). 3 dpf larvae selected for single CSF-cN expression were fixed with 4% PFA for 3 hours and immunostained following standard procedures. Genotyping of the larvae was performed after immunostaining. To assess the connectivity of ventral and dorsal CSF-cNs, the same procedure was followed when the (*pkd2l1-TagRFP*) construct has been injected in the *Tg*(*parg* ^*mnet2*^ *-GFP*)^44^ and *Tg*(*tbx16-GFP*)^46^ *lines* and the (*pkd2l1*:*Gal4*) *with* (*UAS*:*synaptophysin-GFP*)^81^ constructs in the *Tg*(*vglut2a*.*lox-DsRed-lox-GFP*)*line* ^45^. Some of these animals have been imaged live.

### Analysis of the axonal arborization of isolated CSF-cNs

To label and trace individual CSF-cNs, we followed the same procedure as described in ref. 15, in Supplemental Information and in Supplemental Fig. 4.

### Fluorescent *in situ* (FISH)

The *pkd2l1* ISH probe was generated as previously described^13^. The *sst1*.*1* plasmid originates from the Argenton lab, Padova, Italy^52, 82^. *pkd2l1* and *sst1*.*1* plasmids were respectively linearized with NotI and SalI. Digoxigenin (DIG)- and fluorescein (Fluo)-labeled probes were synthesized using SP6 RNA polymerase with the RNA Labeling Kit (Roche Applied Science, Basel, Switzerland) to generate both *pkd2l1* and *sst1*.*1* antisense probes. All probes were purified using the mini Quick Spin RNA Column (Roche, Basel, Switzerland). Whole-mount ISH were performed as previously described^13, 83^ on embryos or larvae fixed in 4% PFA in phosphate buffered saline (PBS) overnight at 4 °C.

### Immunohistochemistry (IHC)

Procedures for IHC were described in Supplemental Information.

### FISH coupled to IHC

Procedures were described in ref. 13. Briefly, *pkd2l1* and *sst1*.*1* FISH were performed prior to IHC of green fluorescent protein (GFP): embryos and larvae were washed and immunostained with the chicken anti-GFP antibody (1:500 dilution, Abcam ab13970, Cambridge, UK) overnight at 4 °C, and then incubated with the corresponding Alexa-conjugated secondary antibodies IgG (1:500, Invitrogen A11039, Carlsbad, CA, USA) combined with DAPI (2.5 μg/mL, Invitrogen D3571, Carlsbad, CA, USA).

### Cell counting

To compare the density of markers investigated, we systematically imaged three regions along the rostrocaudal axis of the fish: segments 3–6 (referred as rostral), 10–13 (referred as middle and displayed in all Figures) and 23–26 (referred as caudal).

### Imaging

Images were acquired using an Olympus FV1000 confocal microscope equipped with a 20 and 40x water and 60X oil immersion objectives using the 405, 473 and 543 nm laser lines or using an upright microscope (Examiner Z1, Zeiss) equipped with a spinning disk head (CSU-X1, Yokogawa) and a modular laser light source (LasterStack, 3i Intelligent Imaging Innovations). To determine the overlap of GFP in the *Tg*(*pkd2l1*:*GCaMP5G*)*icm07* transgenic embryos and larvae with *pkd2l1*, *sst1*.*1* FISH or GABA and 5-HT IHC, fish were mounted laterally in 1, 5% agarose covered of Vectashield Mounting Medium (Vectorlabs, CA, USA). To analyze apical extensions, slices were transferred into Vectashield mounting medium as well (Vectorlab, CA, USA).

### Electrophysiology and optogenetic stimulation

Procedures for optogenetic stimulation of CSF-cNs and electrophysiological recordings of CaP motor neurons are described in Supplemental Information and Supplemental Fig. 7.

### Statistics

We used Student’s t-tests for the morphological comparison of ventral versus dorsal CSF-cNs in WT and *pkd2l1* ^*icm02*/*icm02*^ mutants and two-way ANOVAs to test the interaction between the genotypes and the cells investigated. The level of significance was p < 0.05 for all datasets. p values are represented as the following: (*)p < 0.05; (***)p < 0.001; (****)p < 0.0001.

## Electronic supplementary material

Supplementary Info File #1
