# Supplementary material for: The dual developmental origin of spinal cerebrospinal fluid-contacting neurons gives rise to distinct functional subtypes
Source: Sci Rep. 2017 Apr 7;7:719. doi: 10.1038/s41598-017-00350-1 (PMC5428266; doi:10.1038/s41598-017-00350-1)

The dual developmental origin of spinal cerebrospinal fluid-contacting neurons gives rise to distinct functional subtypes

Lydia Djenoune[1,2], Laura Desban[1], Johanna Gomez[1], Jenna R. Sternberg[1], Andrew Prendergast[1], Dominique Langui[1], Feng B. Quan[1,2], Hugo Marnas[1], Thomas O. Auer[3+], Jean-Paul Rio[1], Filippo Del Bene[3], Pierre-Luc Bardet[1,*], Claire Wyart[1,*]

[1]Sorbonne Universités, UPMC Univ Paris 06, Inserm, CNRS, AP-HP, Institut du Cerveau et de la Moelle épinière (ICM) - Hôpital Pitié-Salpêtrière, Boulevard de l'hôpital, F-75013, Paris, France. [2]Muséum National d'Histoire Naturelle, Paris 75005, France. [3]Institut Curie, Paris 75005, France.

*Correspondence: claire.wyart@icm-institute.org, pierreluc.bardet@icm-institute.org

[+]Present address: Thomas O Auer, Center for Integrative Genomics, Faculty of Biology and Medicine, University of Lausanne, Switzerland.

Supplemental figures legends

Supplemental Fig. 1: Ultrastructure of putative spinal CSF-cNs in wild type zebrafish larva.

(**a**) Transverse section of the spinal cord illustrating multiple cells around the central canal (cc). (**b-d**) Putative ventral CSF-cNs referred to as PVC. (**d**) PVC bearing microvilli (arrowhead) and extending a cilium (arrow) with it apical pole. (**e**, **f**) Putative dorsal CSF-cN referred to as PDC extending microvilli (arrowheads) in contact with the central canal (cc). Scale bars: 10 µm (**a**), 2 µm (**b, c**), 500 nm (**d**), 5 µm (**e**) and 1 µm (**f**).

Supplemental Fig. 2: Use of the APEX2 peroxidase combined with the UAS-Gal4 system and *pkd2l1* specificity to target CSF-cNs.

(**a**) Schematic showing how APEX2 is used to generate diaminobenzidine (DAB) contrast for EM in zebrafish CSF-cNs taking advantage of the UAS-Gal4 system. We injected the *(UAS:APEX2-TagRFP)* construct into *Tg(pkd2l1:Gal4)icm10* fertilized eggs in order to get specific expression of APEX2 in CSF-cNs. After screening for TagRFP expression, larvae were fixed and bathed with a solution of DAB and H_2O_2. APEX2 catalyzes the polymerization and local deposition of DAB, which subsequently attracts electron-dense osmium (OsO_4), giving EM contrast only in APEX2 expressing structures namely CSF-cNs (ventral and dorsal). (**b1**) Lateral view of a *Tg(pkd2l1:Gal4)icm10* larva at 2.5 dpf injected with *(UAS:APEX2-TagRFP)* showing that *pkd2l1* promoter drives APEX2-TagRFP expression in CSF-cNs within the spinal cord. (**b2**) Close up of the rostral spinal cord from a lateral view in the same transgenic animal showing the TagRFP expression in CSF-cNs. (**c**) Co-injection of *(pkd2l1:Gal4)* and *(UAS:APEX2-TagRFP)* within *Tg(pkd2l1:GCaMP5G)icm07* embryos where all CSF-cNs are labeled (green) demonstrates the variegated expression of TagRFP within CSF-cNs (magenta). Note that in this panel, the expression of TagRFP is restricted in some dorsal CSF-cNs. Scale bars: 300 µm (**b1**) and 20 µm (**b2, c**).

Supplemental Fig. 3: DAB deposition in APEX2 expressing CSF-cNs enables EM contrast in the spinal cord.

(**a**) Scheme of a 2.5 dpf larva showing the regions displayed in **b** and **c**. Lateral view of the rostral (**b**) and caudal (**c**) spinal cord of 2.5 dpf *Tg(pkd2l1:Gal4)icm10* larvae injected with *(UAS:APEX2-TagRFP)* and revealed by DAB. Note the DAB^+ CSF-cNs (arrowheads). (**d-e**) Close-ups showing that DAB^+ cells are CSF-cNs characterized by their peculiar apical extension. White line represents the ventral limit of the spinal cord. (**f-g**) Transverse section showing in the spinal cord (SC) the specificity of the DAB deposition in a ventral CSF-cN (arrowhead). NC= notochord. (**g**) Close-up of the dotted rectangle region in (**f**) showing the DAB signal (purple) in a ventral CSF-cN (arrowhead) below the central canal (cc). Scale bars: 100 µm (**b, c**), 50 µm (**d-f**) and 20 µm (**g**).

Supplemental Fig. 4: Characterization of CSF-cNs axonal morphology

(**a**) Projection obtained from z-stacks of a single CSF-cN in a wild-type larva previously injected with *(pkd2l1-TagRFP)*. (**b**) The reconstruction of 3 ventral CSF-cNs (dark blue) and 3 dorsal CSF-cNs (orange) from segment (Seg) 3, 12 and 19 shows that, along the rostrocaudal axis, ventral CSF-cNs are morphologically different from dorsal ones. (**c**) Illustration of the reconstruction of one ventral CSF-cN displays the area covered by the axon and the axonal arborization nomenclature. Horizontal lines represent the limits of the spinal cord and slash dashed lines represent somite boundaries. Vertical black bars represent the dorso-ventral limits of the spinal cord, where the ventral edge corresponds to 0 and the dorsal edge to 1. Scale bars = 20 µm.

Supplemental Fig. 5: The *Tg(pkd2l1:GCaMP5G)icm07* transgenic line recapitulates endogenous *pkd2l1* mRNA expression.

(a-e) FISH for *pkd2l1* (magenta) coupled to GFP IHC (green) on *Tg(pkd2l1:GCaMP5G)* embryos and larvae at 24 hpf (**a**), 48 hpf (**b, e**), 72 hpf (**c**) and 120 hpf (**d**). (**a-d**) Lateral views of the spinal cord from segments 10 to 13 show that all GFP^+ cells (green) in the *Tg(pkd2l1:GCaMP5G)* line

are *pkd2l1*$^{+}$ (magenta). (**e**) Transverse sections of the spinal cord of 48 hpf embryos show the dorso-ventral distribution of a ventral CSF-cN (arrowhead) ventral to the central canal (small dotted circle) and a dorsal CSF-cNs (arrow) above it. (**f**) All CSF-cNs in the *Tg(pkd2l1:GCaMP5G)* line (green) express GABA (magenta). Horizontal lines represent the limits of the spinal cord and slash dashed lines represent somite boundaries. Scale bars = 20µm.

Supplemental Fig. 6: Pkd2l1 is not required for the proper differentiation of spinal CSF-cNs

GABA (**a,** magenta), 5-HT (**b,** magenta) and GFP (**a-b,** green) IHCs on *pkd2l1*$^{icm02/icm02}$ mutants carrying *Tg(pkd2l1:GCaMP5G)icm07* (thus named *Tg(pkd2l1:GCaMP5G)*$^{icm02/icm02}$). (**a**) All CSF-cNs in the mutants express GABA at 72 hpf. (**b**) At 48 hpf, a large proportion of ventral CSF-cNs in the mutants express the 5-HT. Horizontal lines represent the limits of the spinal cord and slash dashed lines represent somites boundaries. Scale bars = 20µm. (**c-e**) *pkd2l1*$^{+/+}$ and *pkd2l1*$^{icm02/icm02}$ ventral CSF-cNs are represented in two shades of orange from dark to light respectively. *pkd2l1*$^{+/+}$ and *pkd2l1*$^{icm02/icm02}$ dorsal CSF-cNs are similarly represented in two shades of blue. (**c**) Counting of the number of ventral and dorsal CSF-cNs in WT and *pkd2l1*$^{icm02/icm02}$ larvae at 3 dpf in three regions of the spinal cord; rostral from segments 3 to 6; middle from segments 10 to 13 and caudal from segments 23 to 26. (**d**) Counting of the number of 5-HT^{+} ventral CSF-cNs in 48hpf WT and *pkd2l1*$^{icm02/icm02}$ embryos. Two-way ANOVAs were performed to test the interaction between the genotypes and the regions were cells were counted. Red lines represent the median and red crosses represent outliers. (**e**) Statistical analysis of 39 WT and 38 *pkd2l1*$^{icm02/icm02}$ ventral CSF-cNs, 15 WT and 23 *pkd2l1*$^{icm02/icm02}$ dorsal CSF-cNs, and 38 *pkd2l1*$^{icm02/icm02}$ ventral and 23 *pkd2l1*$^{icm02/icm02}$ dorsal CSF-cNs comparing the two populations for the axonal arborization area (p ventral WT versus ventral *pkd2l1*$^{icm02/icm02}$ = 0.8192; p dorsal WT versus dorsal *pkd2l1*$^{icm02/icm02}$ = 0.0956; p ventral *pkd2l1*$^{icm02/icm02}$ versus dorsal *pkd2l1*$^{icm02/icm02}$ = 2.2 10^{-5}), the total axon length (p ventral WT versus ventral *pkd2l1*$^{icm02/icm02}$ = 0.7743; p dorsal WT versus dorsal *pkd2l1*$^{icm02/icm02}$ = 0.4241; p ventral *pkd2l1*$^{icm02/icm02}$ versus dorsal *pkd2l1*$^{icm02/icm02}$ = 8.7 10^{-5}), the number of branches (p ventral WT versus ventral *pkd2l1*$^{icm02/icm02}$ = 0.9680; p dorsal WT versus dorsal *pkd2l1*$^{icm02/icm02}$ = 0.8210; p ventral *pkd2l1*$^{icm02/icm02}$ versus dorsal *pkd2l1*$^{icm02/icm02}$

= 0.0242) and the axonal arborization dorso-ventral range (p ventral WT versus ventral *pkd2l1*$^{icm02/icm02}$ = 0.1249; p dorsal WT versus dorsal *pkd2l1*$^{icm02/icm02}$ = 0.1261; p ventral *pkd2l1*$^{icm02/icm02}$ versus dorsal *pkd2l1*$^{icm02/icm02}$ = 2.7 10^{-8}). Each dot represents one cell. Two-sample t-tests to compare two populations were performed for the given parameter.

Supplemental Fig. 7: The monosynaptic connection from CSF-cNs on to CaP primary motor neurons is preserved in the *pkd2l1*$^{icm02/icm02}$ mutant.

(**a**) Experimental Protocol. Fertilized *pkd2l1*$^{icm02/icm02}$ mutant eggs were injected at the one cell stage with both *(pkd2l1:Gal4)* and *(UAS:ChR2-mCherry)* DNA constructs. At 3 dpf, larvae were screened for CSF-cNs expressing ChR2-mCherry with the characteristic basket shape found around CSF-cN-innervated CaPs. Simultaneous optogenetic activation of CSF-cN was performed while performing whole-cell patch clamp recordings from the innervated CaP motor neuron. (**b**) Image of a CSF-cN expressing ChR2-mCherry with characteristic basket formed by CSF-cN axon onto CaP (top), Alexa-filled CaP (middle), merge (bottom). Scale bar= 10 µm. (**c**) Light-mediated activation of the CSF-cN induces a monosynaptic IPSC in the recorded CaP motor neuron in a *pkd2l1*$^{icm02/icm02}$ mutant (top) similarly to WT conditions [1]. Quantification of peak IPSC amplitude in *pkd2l1*$^{icm02/icm02}$ (bottom) shows the same range of values than the ones found in WT [1,2] (n = 2 cells). Each dot represents an IPSC event.

Supplementary Fig. 8: Graphical abstract summarizing the findings of our study.

While all spinal CSF-cNs express *pkd2l1*, this cell population segregates in two types according to their morphology, their neuronal targets (putative V0-v in brown and CaP motor neuron in green) and expression of molecular markers.

Supplemental materials and methods

Immunohistochemistry

For 5-HT IHC, embryos and larvae were fixed in 4% PFA, 1% dimethyl sulfoxide (DMSO), washed, incubated in 50 mM of glycine in 0.1% PBS-Triton X-100 (PBSTx), washed in PBSTx, blocked for 2h in 1% DMSO, 1% NGS, 1% BSA, and 0.7% Triton X-100 and incubated with primary antibody (rabbit anti-5-HT, 1:2000 from Dr Steinbusch, Maastricht University, Netherlands) in blocking buffer overnight at 4°C. After several washes in PBSTx, fish were incubated in corresponding Alexa conjugated secondary antibodies IgG (1:500, Invitrogen, Carlsbad, CA, USA) in blocking buffer overnight at 4°C. Fish were then washed in PBSTx several times before mounting. For TagRFP immunostaining, after 3h of 4% PFA fixation, larvae were washed in successive baths of PBS-Tween 0.1% (PBST). After 1h blocking in 10% normal goat serum (NGS), 1% DMSO, 0.5% Triton X-100 in PBS 1X, we incubated embryos with the primary rabbit anti-TagRFP antibody (1:500, Invitrogen R10367, Carlsbad, CA, USA) overnight at 4°C in a solution of 1% NGS, 1% DMSO, 0.5% Triton X-100 in PBS 1X. After several washes in PBST, larvae were incubated with corresponding Alexa conjugated secondary antibodies IgG (1:500, Invitrogen, Carlsbad, CA, USA) overnight at 4°C and washed several times with PBST. For GABA immunostaining, larvae were fixed for 3h at 4°C in 4% PFA, 0.1% glutaraldehyde, washed in successive baths of PBST, dehydrated stepwise in increasing concentration of methanol and stored for at least two hours at -20°C then rehydrated stepwise. Larvae were digested with 0.5 U/mL dispase (1X, Invitrogen 17105-041, Carlsbad, CA, USA) for 90 min. Larvae were blocked for at least two hours in 2% BSA, 5% NGS, 1% DMSO, PBST and incubated with the primary rabbit anti-GABA antibody (1:2,000, Sigma-Aldrich A2052, St. Louis, MO, USA) overnight at 4°C in a solution of 2% BSA, 2% NGS, 1% DMSO, PBST. After several washes in 1% DMSO, 1% NGS, PBST, larvae were incubated in corresponding Alexa conjugated secondary antibodies IgG (1:500, Invitrogen, Carlsbad, CA, USA) overnight at 4°C. Note that for GABA and 5-HT IHC at 3 dpf, 30% sucrose was added in fixation solutions, fish were treated prior to staining with 0.2 mg/mL collagenase in PBS for 5min and skin was removed with forceps to increase permeabilization.

Analysis of the axonal arborization of isolated CSF-cNs

For each cell, multiple Z-stacks (step size = 1 µm) obtained from a lateral view were acquired to capture the entire axonal arborization. Multiple stacks from a single cell were combined using the Pairwise stitching and Grid/Collection stitching plugins in Fiji [3] or the XuvTools stitching software [4]. The combined files were then exported in Fiji [5] and the cell morphology was reconstructed into a three-dimensional image using the Simple Neurite Tracer (SNT) plugin [6] (**Supplemental Fig. 4b**). The area was measured in MATLAB as the smallest polygon that included all axonal endings (grey area, **Supplemental Fig. 4c**). The total axon length and the number of branches were obtained from SNT (red and purple segments in **Supplemental Fig. 4c**). The traces obtained from SNT were then imported in MATLAB and morphological parameters were extracted using a custom made script available upon request. The dorso-ventral soma positions were measured from the center of the cell body and normalized to the limits of the spinal cord where the ventral edge corresponds to 0 and the dorsal edge to 1 (**Supplemental Fig. 4b, c**). The total axon length was the sum of the length of the main axon and all branches. The minimum dorso-ventral (DV) axon position referred to the most ventral position reached by the axon. The maximum DV axon position referred to the most dorsal position reached by the axon. The DV axonal range was the difference between the two. For illustration purposes, the cell reconstruction images were processed in Photoshop CS5 in order to assign them a color according to their genotype and to display them according to their dorso-ventral index.

Electrophysiology and optogenetic stimulation

Adult homozygous *pkd2l1*$^{icm02/icm02}$ mutants were crossed to obtain *pkd2l1*$^{icm02/icm02}$ mutant offspring. These embryos injected at the one-cell stage with the DNA constructs *(pkd2l1:gal4)* and *(UAS:ChR2-mCherry)* showed very sparse expression of ChR2-mCherry in CSF-cNs. Larvae were pre-screened for fluorescence to identify ChR2-mCherry^{+} CSF-cNs with a characteristic basket structure indicative of a CSF-cN to a CaP motor neuron projection [2]. After recordings, motor neuron identity was confirmed by visualization of Alexa 488 dye and post hoc imaging of ChR2-mCherry. One cell per larva was used for data analysis.

Recordings were performed as described in [1]. Whole cell recording were performed in 3 dpf larvae in artificial CSF (concentrations in mM: 134 NaCl, 2.9 KCl, 1.2 MgCl2, 10 HEPES, 10 glucose, 2 CaCl2, 290 mOsM +/- 3 mOsm, pH adjusted to 7.8 with NaOH). Larvae were pinned through

the notochord with 0.025 mm tungsten pins. Larvae were decapitated to prevent visual light response, and skin and muscle from two segments between segments 8 and 18 were dissected using a glass suction pipette. A MultiClamp 700B amplifier, a Digidata series 1440A Digitizer, and pClamp 10.3 software (Axon Instruments, Molecular Devices 446 Sunnyvale, CA, USA) were used for acquisitions. Patch pipettes (1B150F-4, WPI, Sarasota, FL,USA) with a tip resistance of 8-11 MΩ were filled with internal solution (concentrations in mM: K-gluconate 115, KCl 15, MgCl2 2, Mg-ATP 4, HEPES free acid 10, EGTA 0.5, 290 mOsm, adjusted to pH 7.2 with KOH with Alexa 488 at 10 µM final concentration). Holding potential was -85 mV. Analysis was performed offline using Clampex 10 software (Molecular Devices, California, USA). Activation of a CSF-cN was achieved by whole field stimulation through a 40x objective for 5 ms with a blue LED (UHP-Mic-LED-460, Prizmatic Ltd., Israel, power = 3.88 mW / mm^2) through a Digital Micromirror Device with all mirrors in the "on" position. All experiments were run as sets of 10 trials. Two sets of experiments were performed for each cell.

References

1 Sternberg, J. R. *et al.* Optimization of a Neurotoxin to Investigate the Contribution of Excitatory Interneurons to Speed Modulation In Vivo. *Curr Biol* **26**, 2319-2328, doi:10.1016/j.cub.2016.06.037 (2016).

2 Hubbard, J. M. *et al.* Intraspinal Sensory Neurons Provide Powerful Inhibition to Motor Circuits Ensuring Postural Control during Locomotion. *Curr Biol* **26**, 2841-2853, doi:10.1016/j.cub.2016.08.026 (2016).

3 Preibisch, S., Saalfeld, S. & Tomancak, P. Globally optimal stitching of tiled 3D microscopic image acquisitions. *Bioinformatics* **25**, 1463-1465, doi:10.1093/bioinformatics/btp184btp184 (2009).

4 Emmenlauer, M. *et al.* XuvTools: free, fast and reliable stitching of large 3D datasets. *J Microsc* **233**, 42-60, doi:10.1111/j.1365-2818.2008.03094.xJMI3094 (2009).

5 Schindelin, J. *et al.* Fiji: an open-source platform for biological-image analysis. *Nat Methods* **9**, 676-682, doi:10.1038/nmeth.2019nmeth.2019 (2012).

6 Longair, M. H., Baker, D. A. & Armstrong, J. D. Simple Neurite Tracer: open source software for reconstruction, visualization and analysis of neuronal processes. *Bioinformatics* **27**, 2453-2454, doi:10.1093/bioinformatics/btr390btr390 (2011).

Supplemental Fig. S1

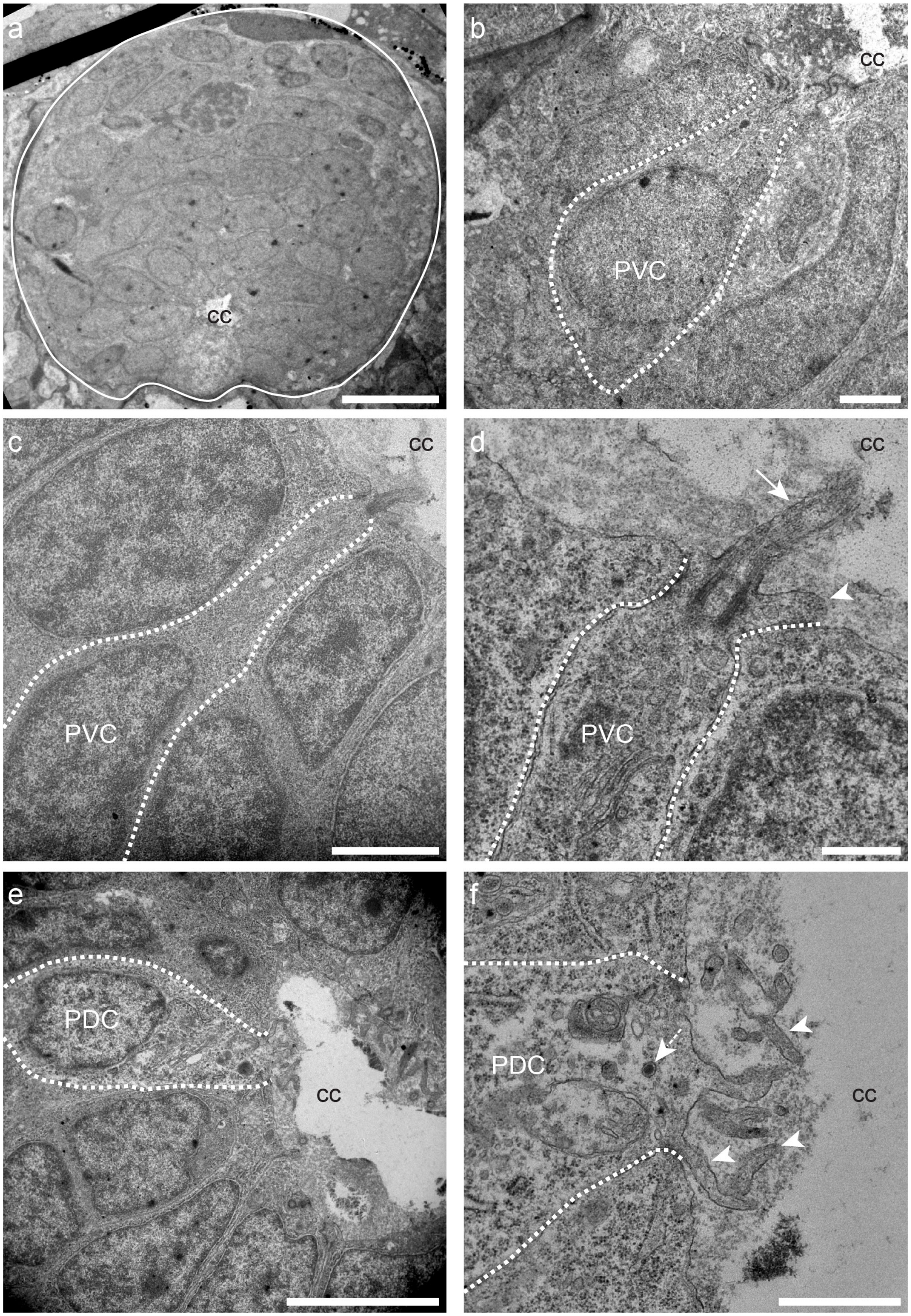

Supplemental Fig. S2

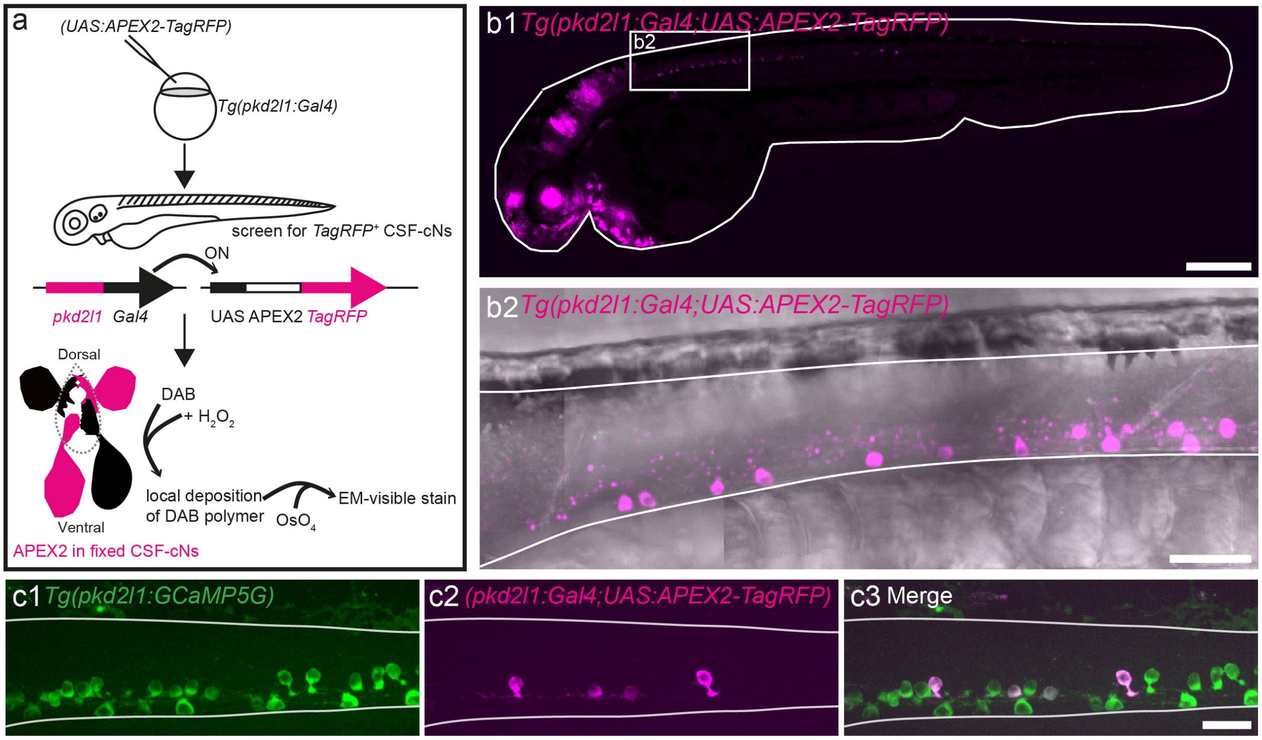

Supplemental Fig. S3

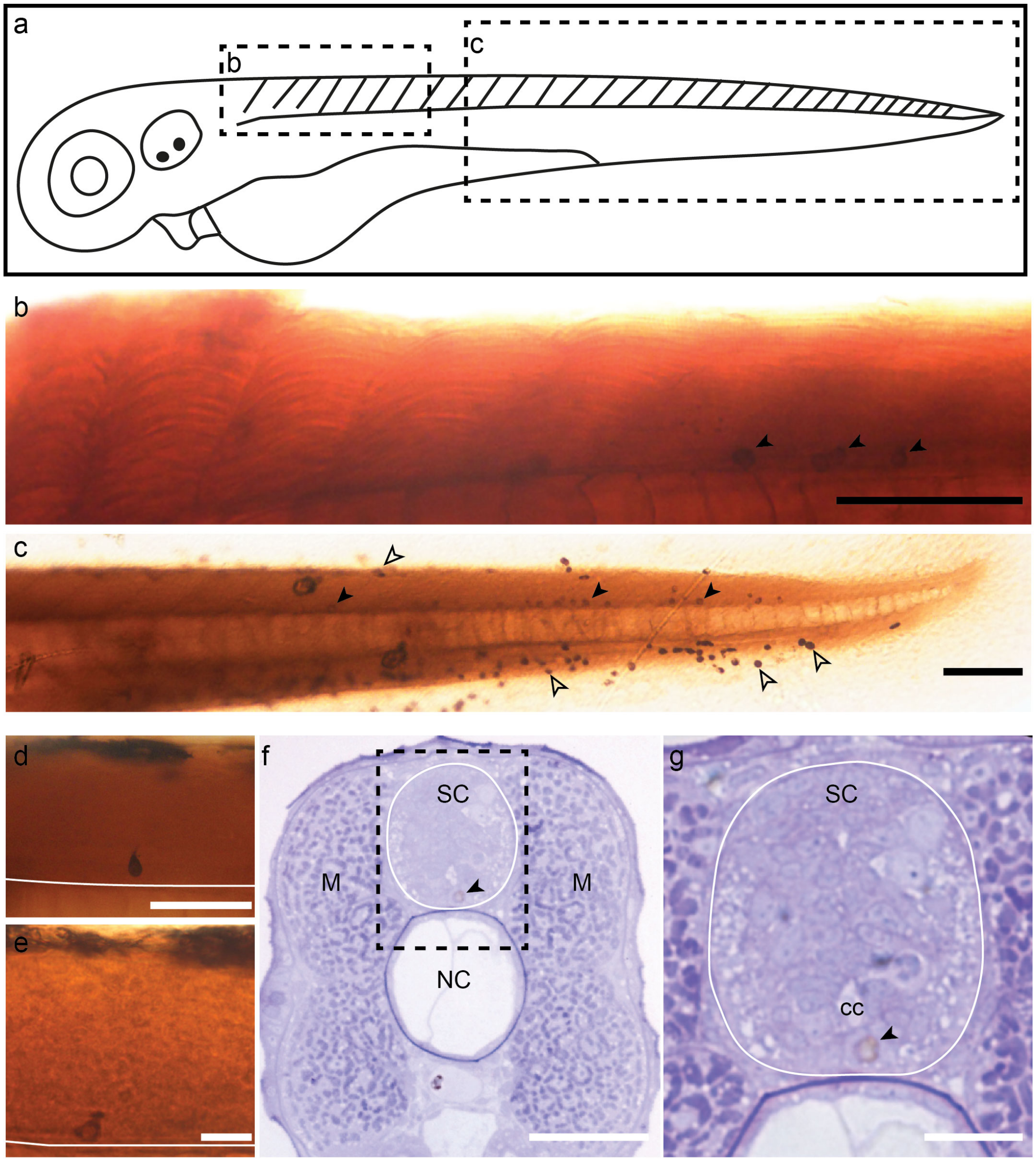

Supplemental Fig. S4

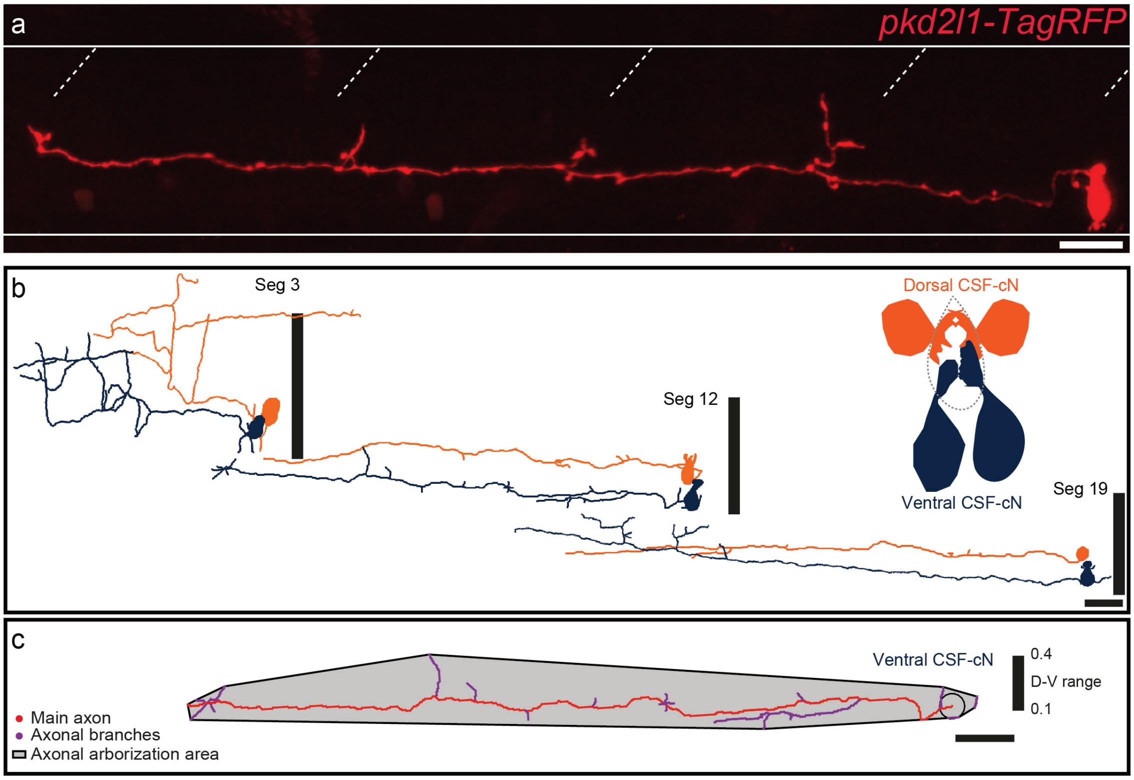

Supplemental Fig. S5

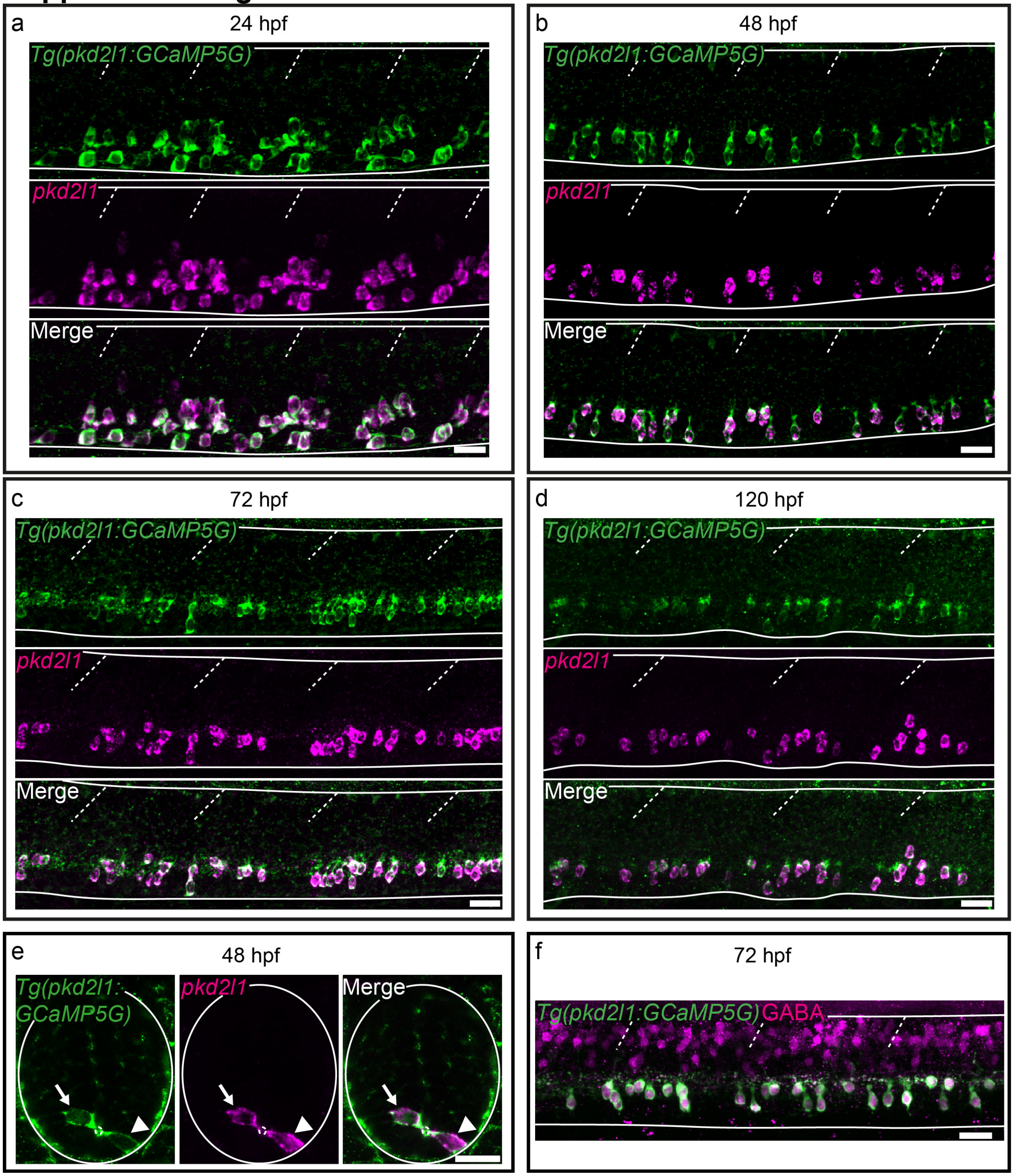

Supplemental Fig. S6

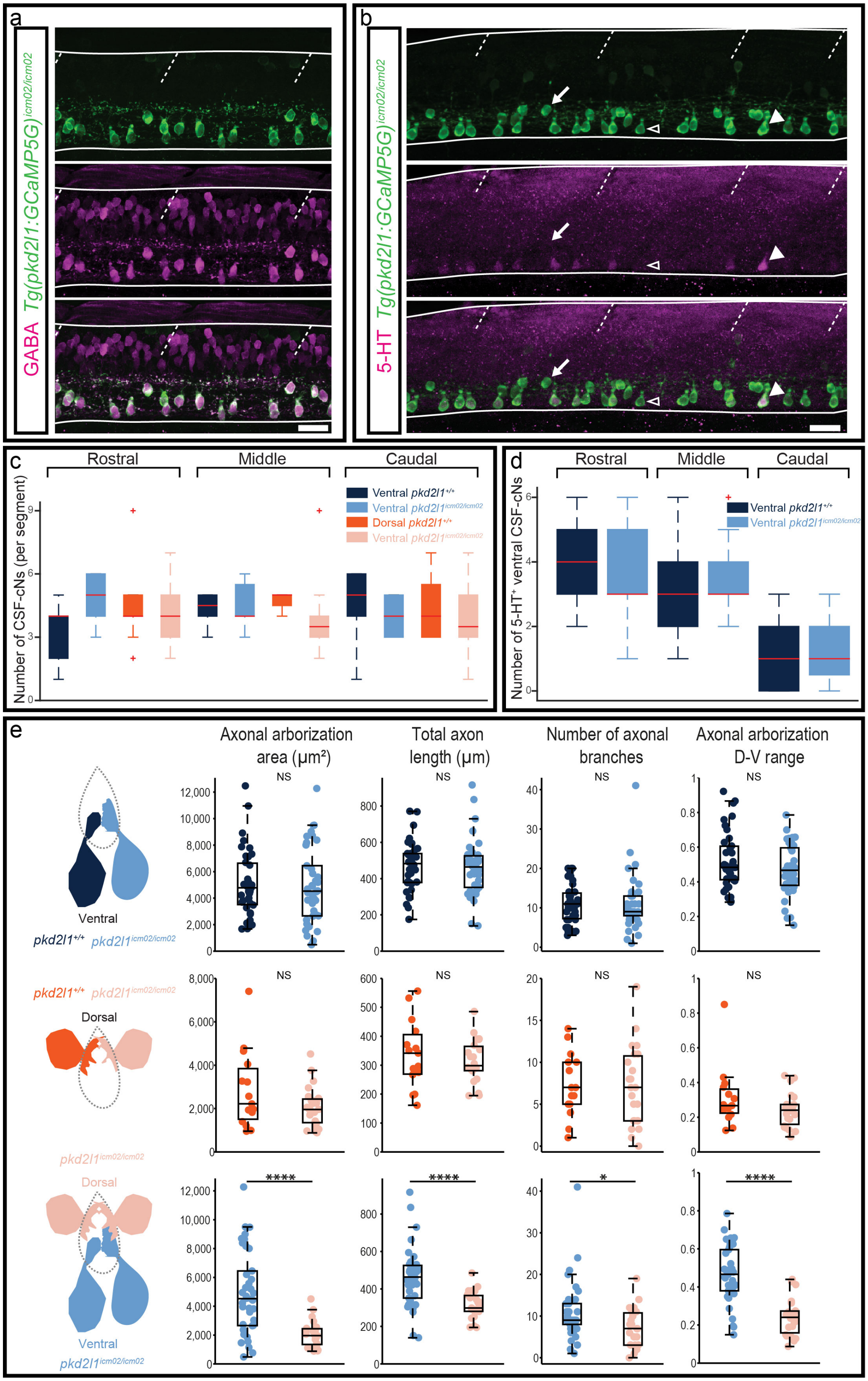

Supplemental Fig. S7

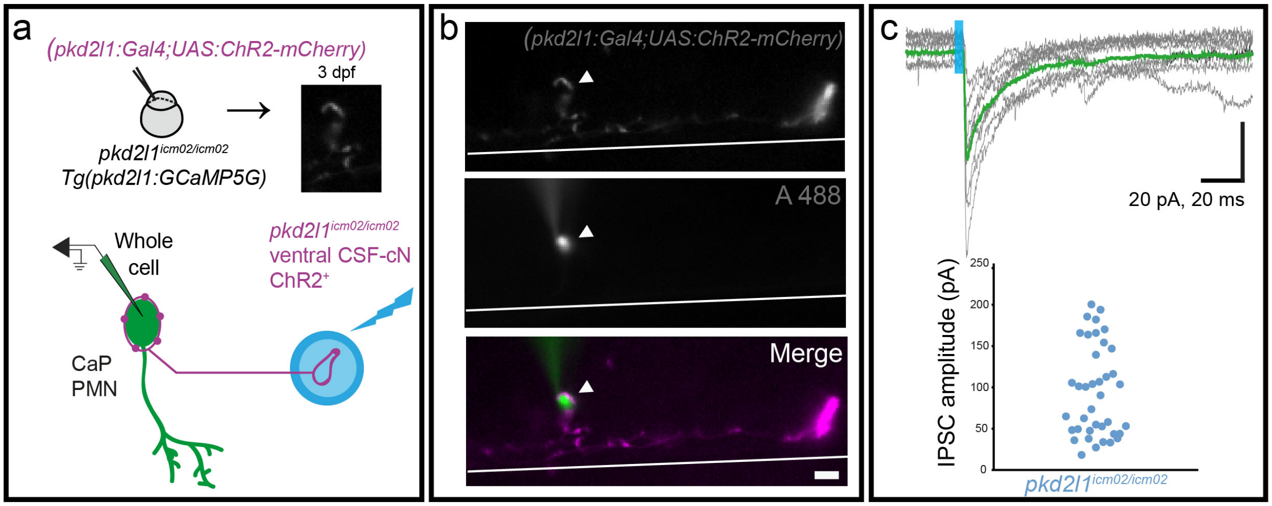

Supplemental Fig. S8

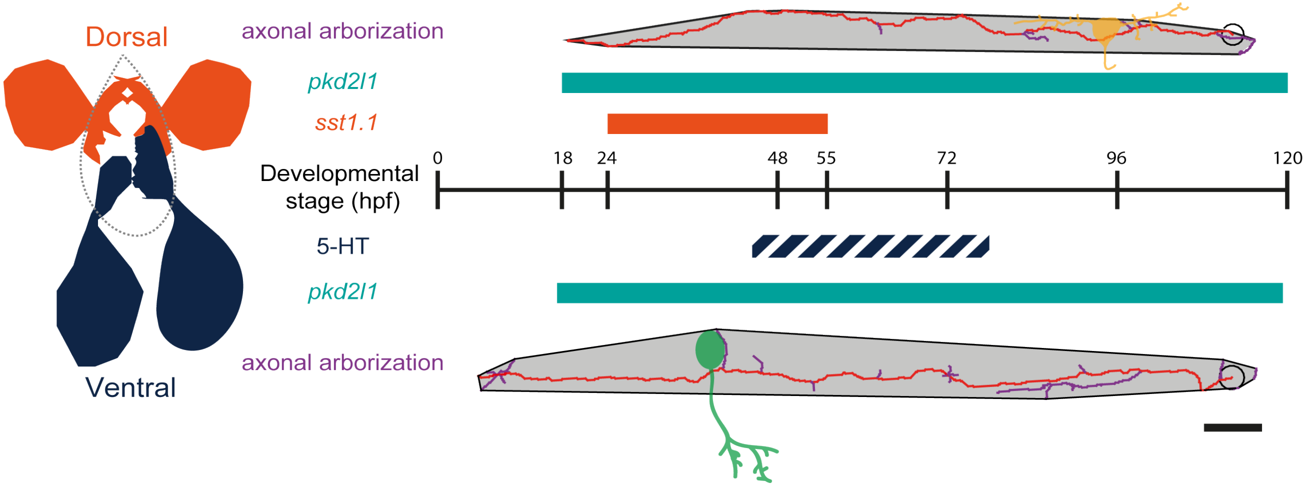

Supplement: Supplementary file 1 — Supplementary Info File #1 [file 41598_2017_350_MOESM1_ESM.pdf]
